# Hospital Appointments

**Published:** 1884-04

**Authors:** 


### Editorial Notes.

Dr. Leidy, of Philadelphia, says: "I have used Churchill's preparation, as made by J. A. McArthur, with the most decided benefit, and am satisfied that a fair trial is all that is required to establish its therapeutic value. I have at this writing several cases in which the syrup is doing beyond my expectations."

We have received from the old Travelers' Insurance Company, of Hartford, a copy of the official engraving of the Bartholdi Statue to be placed in New York harbor. It is the only correct picture of that noble gift, and faithfully represents to the eye the enormous statue, completed and in the midst of its magnificent surroundings.

Emergency hospitals have proved themselves to be a great public benefit. We learn that the authorities of the Sisters' Hospital have purchased the property corner of Michigan and S. Division streets, and propose to erect thereon a commodious and well-appointed building. It would be difficult to find a more desirable location for the hospital than the one selected.

HOSPITAL APPOINTMENTS.—Drs. Davidson, Stockton and Fell have been appointed to serve on the medical staff of the Buffalo Hospital of the Sisters of Charity, and the Emergency Hospital. Drs. H. R. Hopkins and Herman Mynter have accepted positions upon the attending staff of the Buffalo General Hospital. These appointments will meet with general commendation.

THE druggists' war still rages. The cutting of prices seems to be confined to patent medicines and fancy goods. As long as the knights of the pestle do not attempt to make up for their losses on these nostrums by charging our patients more for legitimate drugs and prescriptions, the physician will remain an amused looker-on. We are informed that the sale of patent medicines has largely increased. If this is so, the spectacle of a gullible public purchasing and swallowing more worthless and injurious medicine, because it can be bought at a reduced price, is indeed amusing. There is, however, another and a sad side to it. How much money is wasted and how many lives are lost by a foolish dependence, in the beginning of disease, upon these lying nostrums.

